# Examining the Impact of Aging-Related Mindsets and Motivation on Activity Engagement in Older Adults

**DOI:** 10.1093/geroni/igaa057.1963

**Published:** 2020-12-16

**Authors:** Erica O’Brien, Thomas Hess

**Affiliations:** 1 Pennsylvania State University, University Park, Pennsylvania, United States; 2 North Carolina State University, Raleigh, North Carolina, United States

## Abstract

This study examined short- and long-term patterns of engagement in health-promoting activities due to implicit beliefs about cognitive aging (mindsets) and Need for Cognition (NFC; motivation) in older adults. Prior research suggests higher NFC and growth-oriented mindsets bolster participation by enhancing perceived benefits and minimizing perceived costs of engagement. Survey responses across three bursts of an ongoing longitudinal study (N=678-725 observations) were collected from 148 people aged between 64 and 81 and subjected to three-level multi-level analyses. Results show naturally-occurring, weekly variations in NFC and mindsets that also contribute to short-term variation in activity frequency, diversity, and selectivity. Additionally, NFC and age significantly mediated and moderated the effect of mindsets on some outcomes, respectively. Initial findings highlight the value of taking a dynamic approach and using Selective Engagement Theory to understand activity maintenance. They may also inform efforts to develop interventions that promote healthful behaviors in later life.

